# *In vitro* and *in silico* Studies Reveal *Bacillus cereus* AA-18 as a Potential Candidate for Bioremediation of Mercury-Contaminated Wastewater

**DOI:** 10.3389/fmicb.2022.847806

**Published:** 2022-06-06

**Authors:** Aatif Amin, Muhammad Naveed, Arslan Sarwar, Sunbul Rasheed, Hafiz Ghulam Murtaza Saleem, Zakia Latif, Andreas Bechthold

**Affiliations:** ^1^Department of Microbiology, Faculty of Life Sciences, University of Central Punjab, Lahore, Pakistan; ^2^Department of Biotechnology, Faculty of Life Sciences, University of Central Punjab, Lahore, Pakistan; ^3^Department of Plant Pathology, North Dakota State University, Fargo, ND, United States; ^4^Department of Medical Laboratory Technology, College of Rehabilitation and Allied Health Sciences, Riphah International University, Lahore, Pakistan; ^5^School of Biochemistry and Biotechnology, University of the Punjab, Lahore, Pakistan; ^6^Institute of Microbiology and Molecular Genetics, University of the Punjab, Lahore, Pakistan; ^7^Department of Pharmaceutical Biology and Biotechnology, Institute of Pharmaceutical Sciences, University of Freiburg, Freiburg im Breisgau, Germany

**Keywords:** mercury-biodetoxification, *mer*A, immobilization, industrial wastewater, *in silico*

## Abstract

Mercury (Hg) pollution is a worldwide problem and increasing day by day due to natural and anthropogenic sources. In this study, mercury-resistant (Hg^R^) bacterial isolates were isolated from industrial wastewater of Ittehad Chemicals Ltd., Kala Shah Kaku, Lahore, Pakistan. Out of 65 bacterial isolates, five isolates were screened out based on showing resistance at 30–40 μg/ml against HgCl_2_. Selected Hg-resistant bacterial isolates were characterized as *Bacillus subtilis* AA-16 (OK562835), *Bacillus cereus* AA-18 (OK562834), *Bacillus* sp. AA-20 (OK562833), *Bacillus paramycoides* AA-30 (OK562836), and *Bacillus thuringiensis* AA-35 (OK562837). *B. cereus* AA-18 showed promising results in the resistance of HgCl_2_ (40 μg/ml) due to the presence of *mer*A gene. Scanning electron microscopy (SEM) analysis of immobilized *B. cereus* AA-18 showed the accumulation Hg on the cell surface. The inoculation of immobilized *B. cereus* AA-18 remediated 86% Hg of industrial wastewater up to 72 h at large scale (*p* < 0.05). *In silico* analysis showed structural determination of MerA protein encoded by *mer*A gene of *B. cereus* AA-18 (OK562598) using ProtParam, Pfam, ConSurf Server, InterPro, STRING, Jpred4, PSIPRED, I-TASSER, COACH server, TrRosetta, ERRAT, VERIFY3D, Ramachandran plot, and AutoDock Vina (PyRx 8.0). These bioinformatics tools predicted the structural-based functional homology of MerA protein (mercuric reductase) associated with *mer* operon harboring bacteria involved in Hg-bioremediation system.

## Introduction

Mercury pollution is caused by a number of natural sources like forest fires, volcanic eruptions, nd anthropogenic activities such as coal combustion, mining, manufacturing of chemical compounds, metal processing, incineration, and industrial waste management (Budnik and Casteleyn, 2019). Mercury is a highly toxic heavy metal present in the environment. Mercury and its compounds have a toxic effect on humans and animals, but the pattern of toxicity differs with its chemical states such as organic, inorganic, and metallic (Rice et al., 2014). Mercury is highly bioaccumulative and responsible for neurotoxic and cytotoxic effects in humans (Rahman and Singh, 2019).

Mercury causes protein alterations and loss of function by attaching with sulfhydryl ligands of amino acids (Fashola et al., 2016). It can be transformed into different chemical forms by oxidation–reduction and methylation–demethylation process (Streets et al., 2018). Among all chemical forms of mercury, inorganic mercury ions (Hg^+2^) are highly toxic due to high affinity with cysteine residues of proteins of central nervous system, high lipid solubility, and high uptake rate across biological membranes (Beckers and Rinklebe, 2017). The properties of organic form of mercury also make huge contribution in biomagnifications and bioaccumulations to cause mercury toxicity (Souza-Araujo et al., 2016). Two unique aspects of mercury make it the most toxic environmental contaminant as its huge distribution around the globe and its tendency to get more toxic after methylation (Me-Hg) complex (Jaishankar et al., 2014).

Mercury pollution is a serious global problem as its concentration is increasing day by day in water bodies due to the excess flow of contaminated water containing trace elements and heavy metals (Hsu-Kim et al., 2018). In developing countries including Pakistan, the industrial wastes are directly disposed off into the freshwater without making them harmless or non-toxic (Kirk et al., 2014). Industrial effluents are a massive source of heavy metals, that is, mercury (Hg^2+^), copper (Cu^2+^), nickel (Ni^2+^), cadmium (Cd^2+^), chromium (Cr^3+^), and lead (Pb^2+^) (Ali et al., 2019). Irrigation of crops using such polluted water leads to less productivity of crops and is eventually harmful for human health upon consumption Hg-contaminated foods (Balkhair and Ashraf, 2016). Accumulation of organic and inorganic mercury in the human body causes severe diseases of heart and kidney (Obasi and Akudinobi, 2020).

Various technologies such as immobilization, precipitation, ion exchange, reverse osmosis, and electrochemical treatment have been used for reducing the amount of mercury from industrial wastewater (Zimmerman et al., 2007), but such technologies hold some limitations and drawbacks as being expensive, less effective, basic treatment, and source of hazardous byproducts. Therefore, biodetoxification technique using different bacterial mechanisms is the best possible approach to remediate mercury as it is cost-effective and efficient against all concentrations of heavy metals (Kallithrakas-Kontos and Foteinis, 2016).

Wastewater is treated for detoxifying mercury utilizing bacteria which convert highly toxic form of mercury (Hg^2+^) into less toxic form (Hg^0^). In recent times, bacterial detoxification systems have gained much importance due to their potential to remediate pollutants from environment (Mahbub et al., 2017). Mercury-resistant bacteria harboring *mer* operon have been evaluated for detoxification of mercury compounds. Bacteria use mercuric reductase, a cytoplasmic enzyme encoded by *mer*A gene of operon. Organomercury lyase (*mer*B) is also encoded by *mer* operon that catalyzes the protonolytic breakdown of C-Hg bonds in organomercury compounds. Both *mer*A and *mer*B encode for a periplasmic protein (*mer*P) and various inner membrane proteins such as *mer*T, *mer*C, *mer*E, *mer*F, and *mer*G which help in transporting Hg^2+^ into or out of cytoplasmic membrane (Barkay et al., 2003; Amin and Latif, 2017a).

The utilization of microorganisms with carrier polymers is an appropriate option as it has high productivity, constant quality, and cost-effectiveness. Immobilized cells are reusable with extensive shelf life and protect the cell from external environmental conditions. Immobilized cell system is categorized into four classes on the basis of their physical and support mechanisms, that is, adsorption, aggregation, confinement, and entrapment. Gel entrapment by using natural polymers like agar, alginate, collagen, and gelatin is a technique which can easily be performed under ambient conditions (Martins et al., 2013). Sodium alginate has been used to immobilize bacterial cells to minimize the cell damage (Oyeagu et al., 2018). Microbial cells are entrapped within beads of calcium alginate considered one of the most extensive method used for immobilization of microbial cells (Dong et al., 2017).

Keeping in mind the serious considerations of mercury pollution, the current study is aimed to screen out the Hg-resistant and H_2_S-producing bacteria for their potential role in detoxification of mercury due to the presence of *mer*A gene both in free and immobilized cells. This study also aims to *in silico* study of MerA protein by determining the physicochemical properties, domain and motif analysis, protein–protein interaction and molecular pathway analysis, 2D and 3D structure and ligand-binding prediction, and visualization of interaction between Hg and MerA protein using various computational tools.

## Materials and Methods

### Isolation and Purification of Hg^R^ Bacteria

Samples of industrial wastewater were collected from Ittehad Chemicals Ltd., Kala Shah Kaku, Lahore, Pakistan (Latitude = 31.72°N, Longitude = 74.26°E). The physicochemical properties such as temperature, pH, soluble salts, organic matter, Hg^2+^ concentration, and soluble N, P, and K of all collected samples were measured. A 10-fold serial dilution was prepared for each sample, and from 10^−3^ dilution, 100 μL was poured to LB agar (g/L) plates which contained tryptone, 10; yeast extract, 5; NaCl, 5 and agar; 15. LB media were supplemented with different concentrations ranging from 30 to 40 μg/ml HgCl_2._ Plates were incubated for 24 h at 35°C. After incubation, bacterial colonies having different morphological characteristics were observed and purified on new LB plates containing HgCl_2._

### Morphological and Biochemical Characterization of Hg^R^ Bacterial Strains

Hg^R^ bacterial strains AA-16, AA-18, AA-20, AA-30, and AA-35 were analyzed for various morphological and biochemical characteristics, that is, Gram staining, spore staining, mannitol fermentation, catalase, citrate, oxidase, nitrate reductase, Voges–Proskauer, H_2_S production, motility, and gelatin hydrolysis tests were performed for the determination of their approximate genus.

### Molecular Characterization of 16S rRNA and *mer*A Genes

Bacterial isolates AA-16, AA-18, AA-20, AA-30, and AA-35 showing high resistance against HgCl_2_ were identified by 16S rDNA ribotyping. Genomic DNA of selected bacterial isolates was extracted by WizPrepTMg DNA mini kit (https://www.wizbiosolution.com/). PCR amplification of selected genes was performed using primers for 16S rRNA gene; 16S-F (5′AGAGTTTGATCCTGGCTCAG3′) and 16S-R (5′AAGGAGGTGATCCAGCCGCA3′) (Normand, 1995) and for *mer*A gene; *mer*A-F (5′ATGACCACCCTGAAAATCAC3′) and *mer*A-R (5′AAAGCACGACGCGGCCTACAT3′). Amplification conditions were optimized for selected genes as denaturation at 94°C for 1 min, annealing at 55°C for 1 min, extension at 72°C for 2 min, and chain elongation at 72°C for 10 min, for 35 cycles. Amplified PCR products were sent to the Macrogen sequencing core facility (https://dna.macrogen.com/) in Korea. The obtained sequences were subjected to BLASTn for homology analysis and submitted to GenBank.

### Phylogenetic Analysis

Phylogenetic analysis among bacterial species of the *Bacillus* genera on the basis of 16S rRNA and *mer*A genes was done by multiple sequence alignment through ClustalW. MEGE-X software, ver.05 (https://www.megasoftware.net/), was used to construct phylogenetic tree by neighbor-joining method. Bootstrap test value with 1000 replicates was used to determine the percentage homology among different species.

### Detoxification of Hg^2+^ by Selected Bacterial Isolates

Detoxification of toxic ionic mercury (Hg^2+^) into less elemental mercury (Hg^0^) was carried out by dithizone method (Elly, 1973). Briefly, 5 μL culture of each Hg^R^ bacterial strains AA-16, AA-18, AA-20, AA-30, and AA-35 was inoculated in 5 ml of LB medium and incubated at 37 °C for 24 h. From each culture (O.D. 2.0 at 600 nm), 1.5 ml was inoculated in six flasks (6th flask was taken as negative control) containing 30 ml of LB medium supplemented with 40 μg/ml HgCl_2._ All the flasks were put to shaking incubator at 120 rpm for 24 h at 37 °C. Conc. H_2_SO_4_ was used to adjust pH 4.0 of each culture medium. All the cultures were centrifuged at 12,000 × g for 15 min, and supernatant was taken and transferred to separating funnel. Supernatant was allowed to cool, and 2.5 ml of chloroform and 4 ml of 6N acetic acid were added with continuous shaking. Dithizone solution, 5 ml of 0.001%, was added to remaining solution and agitated for 1 min. Cotton was put to the tip of separating funnel to separate the layers, and dithizone–Hg complex was eluted. Selected Hg^R^ bacterial strains underwent reduction reaction (Hg^2+^ Hg^0^) by vaporizing Hg^0^ through mercuric reductase. No significant color was produced with dithizone on reduction of given concentration of Hg^2+^ to Hg^0^. Orange color was observed in the presence of Hg^2+^, and color became more intense with higher concentration of Hg^2+^. The detoxification of LB medium was estimated by the evaluation of optical density (O.D.) of water-free chloroform extracts of each strain at 500 nm against blank.

### Immobilization of Hg^R^ Bacterial Cells

Cultures of Hg^R^ bacterial strains: *B. cereus* AA-18 was grown at 35°C overnight in 50-ml LB medium at 150 rpm. Culture was centrifuged at 12,000 × g for 5 min at room temperature when their O.D._600nm_ was 1.0. Encapsulation of cultures was performed with 2% sterile solution of sodium alginate containing 3% sucrose. Bacterial–alginate mixture was extruded dropwise from a height of approximately 15 cm into an access of 75 mM of CaCl_2_ with constant stirring. Bacterial cells entrapped with the beads of calcium alginate were indurated in CaCl_2_ solution within 15 min. Solution of 5 mM CaCl_2_ was used to wash synthetic encapsulated beads for 1 h. Immobilized beads were collected by filtration and stored at 4°C for prolonged use.

### Detoxification of Hg^2+^ by Immobilized Bacterial Cells

Detoxification of Hg^2+^ by immobilized bacterial cells was performed by inoculation of 4-g synthetic beads in 50-ml LB medium supplemented with 30 μg/ml HgCl_2_. Culture was filtered and washed with sterile 5 mM CaCl_2_ after every 72 h at 30 °C. For estimation of Hg^2+^, dithizone method was performed. Free cell culture of same strain was also grown in LB medium and used as a negative control.

### Shelf Life of Immobilized *B. cereus* AA-18

Four aliquots of immobilized cells were used to determine their shelf life. Each aliquot was grown in LB medium containing 20 μg/ml HgCl_2_ at 30°C for 48, and amount of Hg^2+^ was quantified in bead-free extract by dithizone method.

### Bioremediation of Wastewater by Immobilized AA-18 and SEM Analysis

Large-scale experiment was set up to determine the ability of immobilized AA-18 to remove Hg^2+^ from wastewater effluent up to 12 days. Three containers were taken. In first container, 15 L of distilled water was taken along with 3 L of immobilized AA-18 cells. In the second container, 15 L of industrial wastewater and 3 L of free *B. cereus* AA-18 cells grown to log phase were taken. In the third container, only 15 L of industrial wastewater was taken. Samples were incubated up to 10 days at 37 °C. Samples were spun at 5,000 × g for 5 min to separate the cells. Supernatant was used for the quantification of Hg^2+^ in industrial wastewater removed by bacterial cells. SEM analysis of immobilized *B. cereus* AA-18 was performed by diluting one drop of 48 h culture with 15 ml of ddH_2_O. Sample was critically point dried on a carbon surface slide. The morphological, topographical, and crystallographic information about bacterial cell was provided by scanning electron microscope (SEM) (JEOL-JSM-6480).

### Statistical Analysis

All experiments were performed independently in triplicates, and the experimental data were subjected to mean, standard deviation, and analysis of variance (ANOVA) using statistical package SPSS (SPSS Inc., Chicago, USA).

### Physicochemical Characterization of MerA Protein

Portparam tool was used for the physiochemical analysis of MerA protein. Portparam (https://web.expasy.org/protparam/) gave results on the basis of protein information stored in Swiss-Port, TrEMBL, and the query sequence used by the user as input. Parameters like molecular weight, estimated half-life, amino acid composition, extinction coefficient, instability index, atomic composition, aliphatic index, and grand average of hydropathicity were evaluated.

### Domain and Motif Analysis

Conserved sequence and functional analysis of MerA protein were predicted using Pfam, ConSurf Server, and InterProScan. Pfam (http://pfam.xfam.org/) works on the basis of its huge collection of protein families processed by multiple sequence alignment and hidden Markov model (HMM) (Finn et al., 2016). Functional domains and important sites of the protein sequence were functionally predicted and analyzed by InterProScan (https://www.ebi.ac.uk/interpro/search/sequence/). For the purpose of protein subcellular localization and binding domains, prediction Motif Finder tool was used (https://www.genome.jp/tools/motif/).

### Protein–Protein Interaction and Molecular Pathway Analysis

STRING database contains information regarding every possible contact among the connected proteins. MerA protein interactions with other protein were identified using STRING (https://string-db.org/). This database was enabled to predict protein interactions in various organisms (Szklarczyk et al., 2021). Kyoto Encyclopedia of Genes and Genomes (KEGG) software (https://www.genome.jp/kegg/pathway.html) was used for high understanding of functional activity of *mer*A gene and its role in biological systems. KEGG is a collection of different databases which is overloaded with information regarding genomes, diseases, drugs, chemical substances, and metabolic pathways (Kanehisa et al., 2017).

### 2D Structural Prediction of MerA

Secondary structure prediction of selected MerA protein was performed using PSIPRED tool (http://bioinf.cs.ucl.ac.uk/psipred/). It determined the secondary structure of protein on the basis of PSI-BLAST position-dependent matrices. The query sequence for the prediction was provided in the form of protein FASTA sequence or FASTA file.

### 3D Structural and Ligand-Binding Site Prediction

TrRosetta (https://yanglab.nankai.edu.cn/trRosetta/) was used for the prediction of 3D structure of MerA protein. It is online software for secondary and tertiary modeling of proteins. Input was provided in the form of a query sequence consisting of single amino acid sequence or a multiple sequence alignment. Generally, choice of homologous protein template is beneficial for accurate model formation. TM-score indicated the accuracy of the 3D structure of protein. TM-score ranges from 0 to 1, and acceptable range is above 0.5. Computed atlas of surface topography of proteins (CASTp) was used for the confirmation of binding pockets available in the protein model.

### 3D Structural Verification

3D structures of our protein were validated by ERRAT, VERIFY 3D, and Ramachandran plot. ERRAT (http://servicesn.mbi.ucla.edu/ERRAT/) analyzed the binding interactions of different amino acids at the atomic level. It also generated a validation score on the basis of statistical evidence. Ramachandran plot (https://saves.mbi.ucla.edu/) validated the protein 3D structure by the torsional angles—phi (ϕ) and psi (ψ)—of amino acid residues present in peptide chain. It gave an estimate of disallowed and allowed torsional angles and served as a significant parameter to assess protein 3D model.

### Mercuric Compounds

Highly toxic Hg^2+^ compounds found in nature were searched in the literature and downloaded their 2D structures in (.sdf) format from PubChem (https://pubchem.ncbi.nlm.nih.gov/). Using the 2D structure of Hg^2+^ compounds available in (.sdf) format, their 3D structures in (.pdb) format through OpenBabel-3.1.1 tool were generated. All the Hg^2+^ compounds used in this study contained toxic properties and possessed threats for the environment and the biological life.

### Docking

Molecular screening of organic and inorganic Hg^2+^ compounds was performed by AutoDock Vina and PyRx tool. During this process, Hg^2+^ molecules were docked against the MerA protein. PyRx is an offline tool which allowed to virtually screen out multiple ligands for computational drug designing or to check the interaction between molecules and the targets. For visualization of interactions between Hg^2+^ and MerA, PyMol and Discovery Studio were used. The results in 3D representation made it easier to understand the interactions at molecular level.

## Results

### Screening, Morphological, and Biochemical Characterization Hg^R^ Bacterial Isolates

Selection of bacterial strains was based on Hg^2+^ resistance potential at different concentrations, that is, 30–40 μg/ml HgCl_2_. The physicochemical parameters of selected samples at the time of collection were determined as follows: temperature 25 °C, pH 6.2, soluble salts 0.65 g/Kg, organic matter 7.9 g/Kg, Hg^+2^ concentration 30–40 μg/ml, soluble N, P, and K 50.4, 4.0, and 43.5 mg/Kg. Four AA-16, AA-20, AA-30, and AA-35 out of 65 bacterial isolates were selected based on showing minimal inhibitory concentration (MIC) value at 30 μg/ml, whereas the strain AA-18 showed highest resistance against HgCl_2_ up to 40 μg/ml. *Bacillus megaterium* strain MB1 was taken as positive control on the basis of showing highest resistance up to 40 μg/ml against HgCl_2_ as shown in Table 1. Selected bacterial strains were morphologically and biochemically characterized as *Bacillus* spp. (Table 2).

**Table 1:** Bacterial isolates showing resistance at different concentrations of HgCl_2_.

**Bacterial isolates**	**Growth against HgCl**_**2**_					
	**30 μg/mL**	**32 μg/mL**	**34 μg/mL**	**36 μg/mL**	**38 μg/mL**	**40 μg/mL**
AA-16	+	+	+	-	-	-
AA-18	+	+	+	+	+	+
AA-20	+	+	+	-	-	-
AA-30	+	+	+	-	-	-
AA-35	+	+	+	-	-	-
MB1	+	+	+	+	+	+

**Table 2:** Morphological and biochemical characterization of selected bacterial isolates.

	**Bacterial isolates**				
**Morphology and biochemical tests**	**AA-16**	**AA-18**	**AA-20**	**AA-30**	**AA-35**
Gram staining	Rods	Rods	Rods	Rods	Rods
Spore	+	+	+	-	+
Mannitol fermentation	+	-	-	+	-
Catalase	+	+	+	+	+
Citrate	+	+	+	-	+
Oxidase	+	-	-	+	-
Nitrate reductase	+	+	+	+	+
Voges Proskauer	+	+	+	+	+
H_2_S production	-	+	-	-	-
Motility	+	+	+	-	+
Gelatin hydrolysis	+	-	-	+	+
Bacterial isolates	*Bacillus* sp.	*Bacillus* sp.	*Bacillus* sp.	*Bacillus* sp.	*Bacillus* sp.

### Molecular Characterization of 16S rRNA and *mer*A Genes

Selected bacterial strains (AA-16, AA-18, AA-20, AA-30, and AA-35) showing resistance against HgCl_2_ were characterized by 16S rDNA ribotyping and identified as *B. subtilis* AA-16 (OK562835), *B. cereus* AA-18 (OK562834), *Bacillus* sp. AA-20 (OK562833), *B. paramycoides* AA-30 (OK562836), and *B. thuringiensis* AA-35 (OK562837).

**Figure 1:**
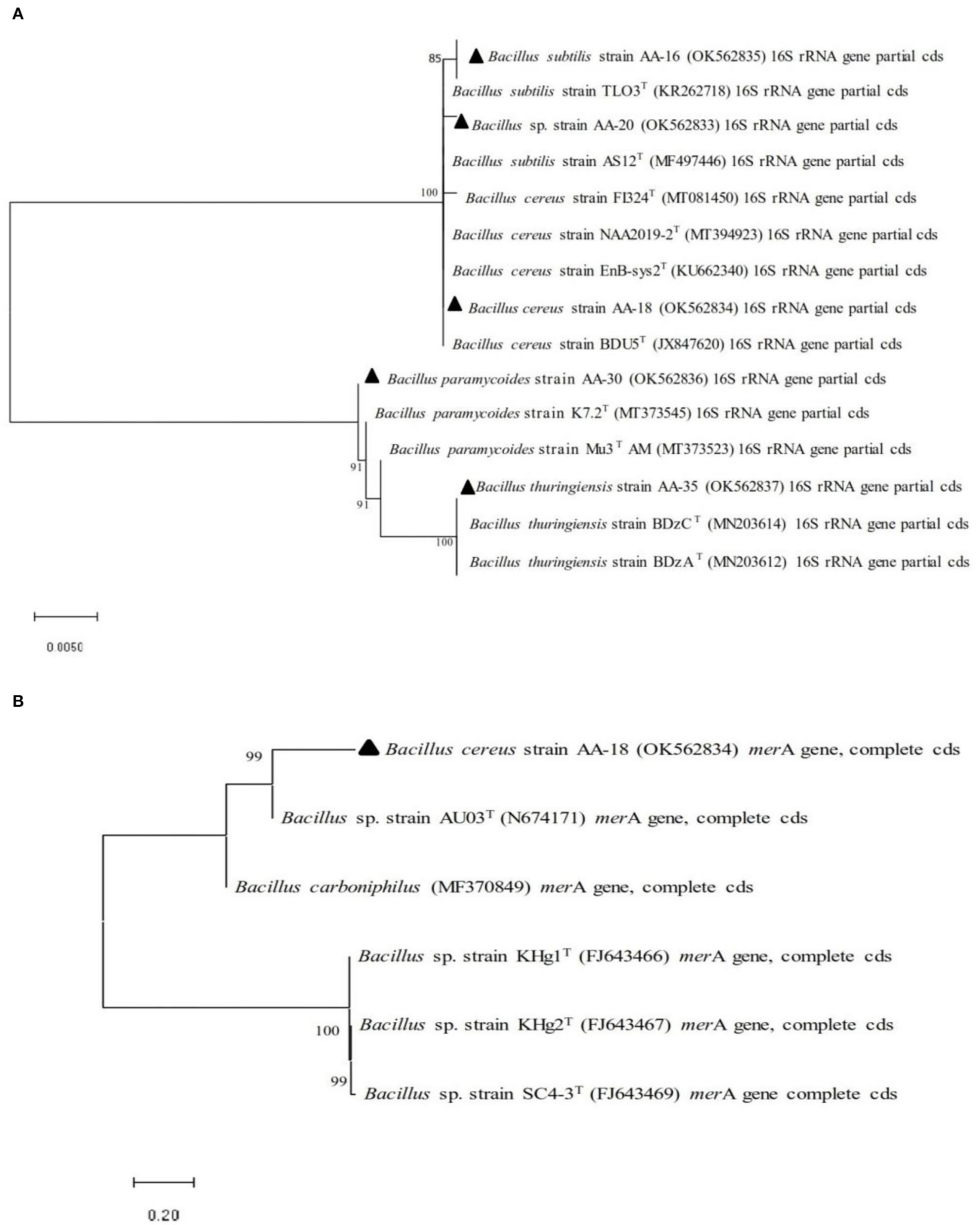
Phylogenetic analysis performed by neighbor-joining method of selected Hg-resistant bacterial strains with other closely related species based on **(A)** 16S rRNA gene sequence and **(B)** *mer*A gene sequence.

Selected bacterial strains (AA-16, AA-18, AA-20, AA-30, and AA-35) on the basis of 16S rRNA sequencing were analyzed by constructing phylogenetic tree and compared with the sequences of type strains (https://lpsn.dsmz.de/). Phylogenetic analysis revealed 85% homology of selected strain AA-16 with type strain, *B. subtilis* TL03^T^ (KR26718). Similarly, strain AA-20 showed 100% homology to other type strains, *B. subtilis* AS12^T^ (MF497446), and other closely related species. Selected strain AA-18 was found to be 100% homologous with *B. cereus* BDU5^T^ (JX84760). In the next clade, strain AA-30 showed 100% similarity among *B. paramycoides* K7.2^T^ (MT373545) and Mu3AM^T^ (MT373523). Similarly in clade 5, selected strain AA-35 showed 100% homology with *B. thuringiensis* BDzC^T^ (MN203614) and other closely related species as shown in Figure 1A.

Selected Hg^R^ bacterial strains were confirmed for the presence of *mer*A gene which encodes mercuric reductase (MerA), an enzyme involved in the conversion of toxic ionic mercury (Hg^2+)^ into less toxic elemental mercury (Hg^0^). Phylogenetic analysis based on *mer*A gene revealed 100% homology among selected Hg^R^ bacterial strain and other closely related species (Figure 1B).

### Estimation of Hg^2+^-Detoxification by Selected Bacterial Isolates

**Figure 2:**
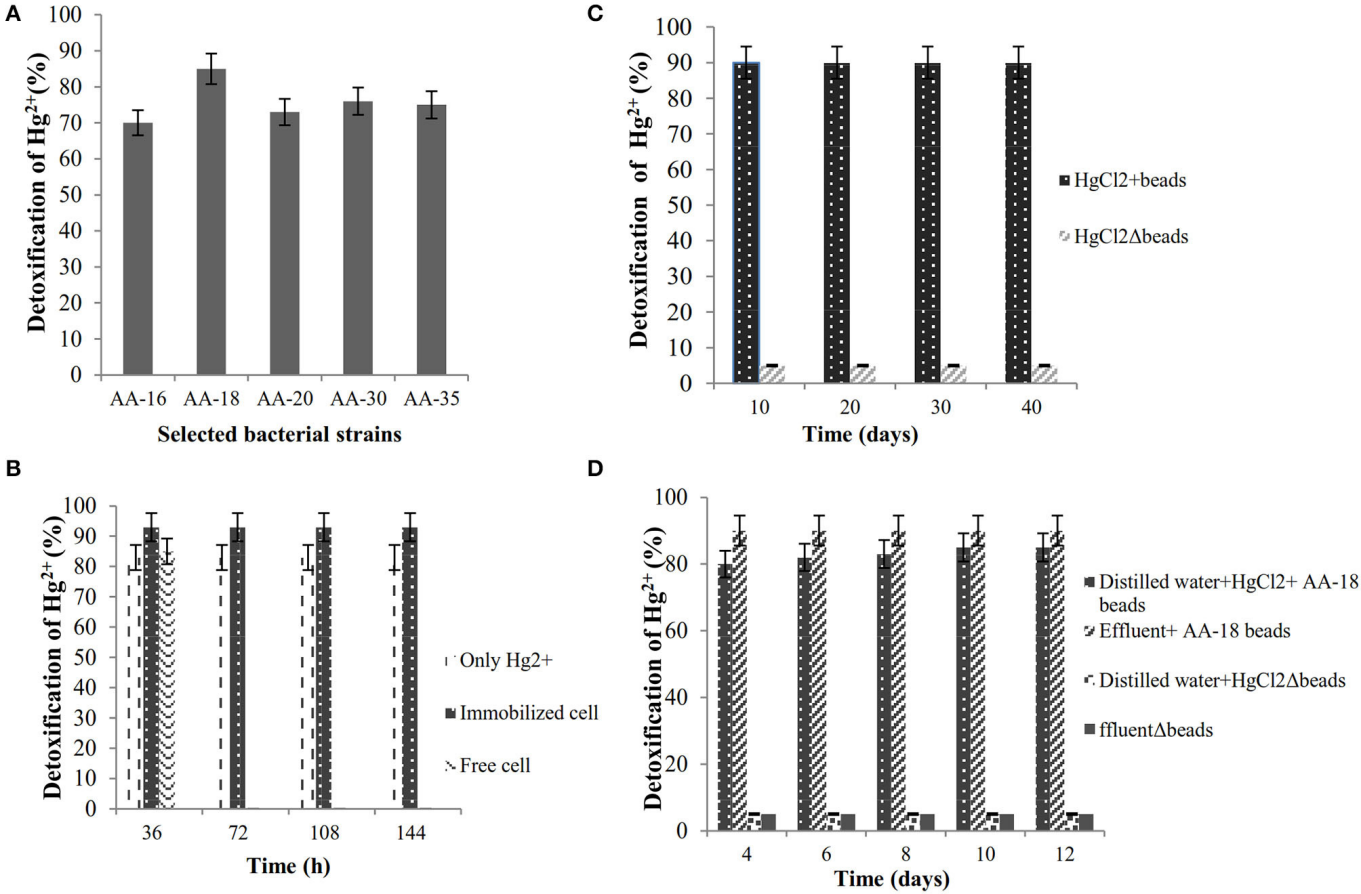
**(A)** Biodetoxification of Hg^2+^ by selected bacterial isolates in LB medium. **(B)** Biodetoxification of Hg^2+^ by immobilized strain AA-18. **(C)** Shelf-life analysis of immobilized *B. cereus* AA-18. **(D)** Bioremediation of Hg^2+^ polluted wastewater by immobilized AA-18.

The ability of selected Hg^R^ bacterial isolates to detoxify Hg^2+^ in LB medium containing 30 μg/ml HgCl_2_ was evaluated by dithizone method. The results showed that *B. cereus* AA-18 possessed the highest ability to remediate Hg^2+^ up to 85% from the medium and other selected strains, AA-16, AA-20, AA-30, and AA-35 detoxified 70, 73, 76, and 75%, respectively, (*p* < 0.05) as shown in Figure 2A.

### Bioremediation of Hg^2+^ by Immobilized AA-18

Highly Hg-resistant *B. cereus* AA-18 was immobilized as synthetic beads in sodium alginate and remediated 28 μg/ml (93%), whereas free cells detoxified 26 μg/ml (85%) of Hg^2+^ from LB medium supplemented with 30 μg/ml HgCl_2_ (*p* < 0.05) as shown in Figure 2B. Immobilized beads of *B. cereus* AA-18 dissolved gradually, but their potential to remediate Hg^2+^ remained same upon using them in four cycles. SEM analysis of sodium alginate immobilized *B. cereus* AA-18 showed the accumulation of Hg on the cell surface due to production of H_2_S which reacted with Hg^2+^to form HgS.


$$Hg^{2+}\;+\;H_{2}S\to HgS\;+\;2H^{+}$$

### Shelf Life of Immobilized *B. cereus* AA-18

Synthetic beads of *B. cereus* AA-18 were stored at 4°C for 2 months, and their detoxification kinetics was observed up to 40 days with an interval of 10 days. The results showed that ability to detoxify Hg^2+^ by AA-18 remained same even after storing for 2 months as shown in Figure 2C. After every interval of 10 days, two hundred entrapped cells (8g) were added in 100-ml LB medium supplemented with 30 μg/ml HgCl_2_ and incubated at 37°C for 48 h. The amount of Hg^2+^ was quantified by dithizone method in the residual cell free extract.

### Bioremediation of Hg^2+^ in Wastewater by Immobilized AA-18 and SEM Analysis

**Figure 3:**
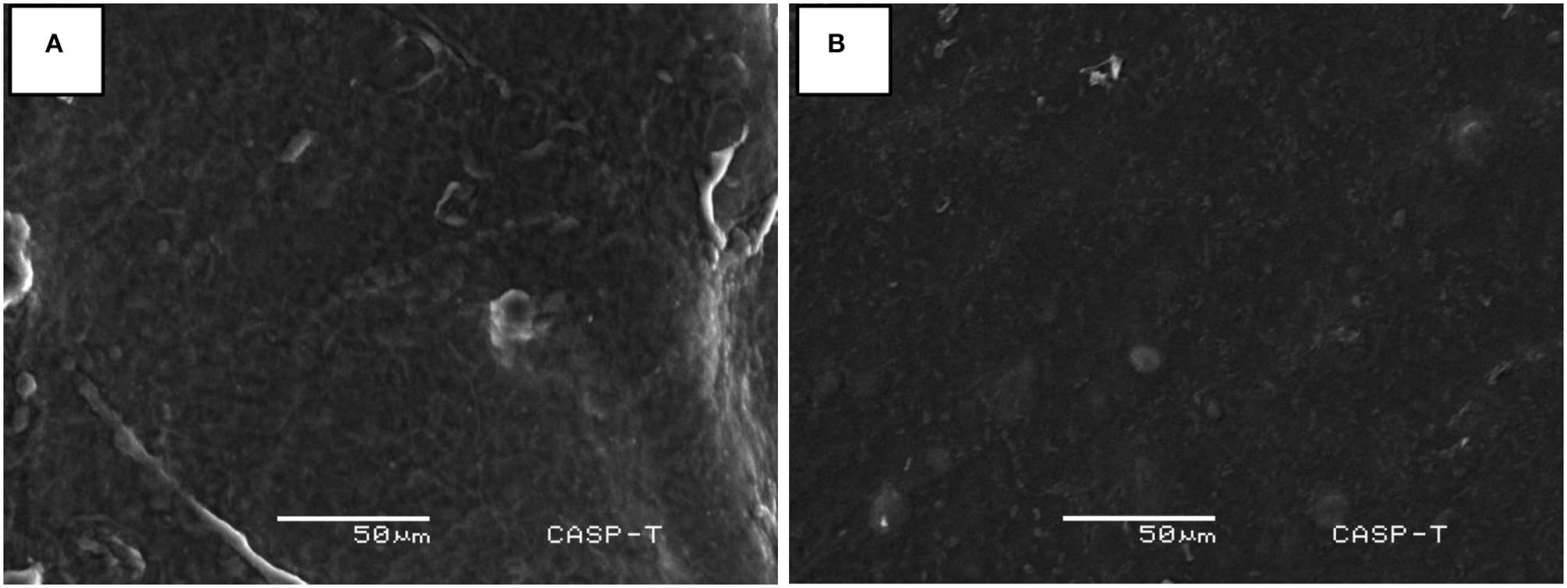
SEM analyses of Hg^2+^ detoxification in industrial wastewater **(A)** with beads of immobilized *B. cereus* AA-18 and **(B)** without beads of immobilized AA-18.

An experiment was designed in laboratory to evaluate the potential of immobilized *B. cereus* AA-18 to detoxify Hg^2+^ up to 12 days. The results revealed that removal of Hg^2+^ by immobilized AA-18 was higher in industrial effluent than distilled water due to the presence of other heavy metals in effluent that acted as essential cofactors such as cobalt (Co^2+^), copper (Cu^2+^), iron (Fe^2+^), manganese (Mn^2+^), and zinc (Zn^2+^). These microbes along with other cofactors speeded up the Hg^2+^ removal from industrial effluent. Therefore, distilled water with immobilized AA-18 detoxified 35% of Hg^2+^ after 12 days whereas 90% of Hg^2+^ was remediated from industrial effluent by immobilized AA-18 (*p* < 0.05) as shown in Figure 2D. A crystalline structure of HgS deposited on the cell membrane of *B. cereus* AA-18 confirmed its ability to remediate mercury in contaminated wastewater as shown in Figure 3.

### Conservedness of *mer*A Gene

Pfam analysis explained that *mer*A gene contained a conserved domain of amino acids which is a heavy metal association (HMA) domain. This domain comprised of two conserved residues of cysteine which enabled it to bind with heavy metals. CoSurf provided a 3D representation of conservedness in selected MerA protein along with a color scale showing degree of conservedness. Supplementary Figure S1 depicted the degree of conservedness occupied by the amino acid sequence.

### Physiochemical Properties and Functional Analysis

Physiochemical analysis of *mer*A gene sequence showed 561 amino acids with molecular weight of 58,767.07 in the sequence. The instability index of MerA protein was 33.33 which predicted it a stable protein. GRAVY hydropathicity was 0.084, and half-life was predicted as 30 h in mammalian reticulocytes, >20 h in yeast cell, and >10 h in *Escherichia coli*. MOTIF tool and InterProScan functionally characterized the gene on the basis of certain domains present in *mer*A gene such as pyridine nucleotide-disulfide oxidoreductase, heavy-metal-associated domain, FAD-binding domain, glucose-inhibited division protein A, NAD(P)-binding Rossmann-like domain, and family of unknown function (DUF6286). Biological role of MerA was determined by InterProScan as detoxification of Hg^2+^ ion and cellular redox homeostasis.

### Protein-Protein Interactions and Molecular Pathways

**Figure 4:**
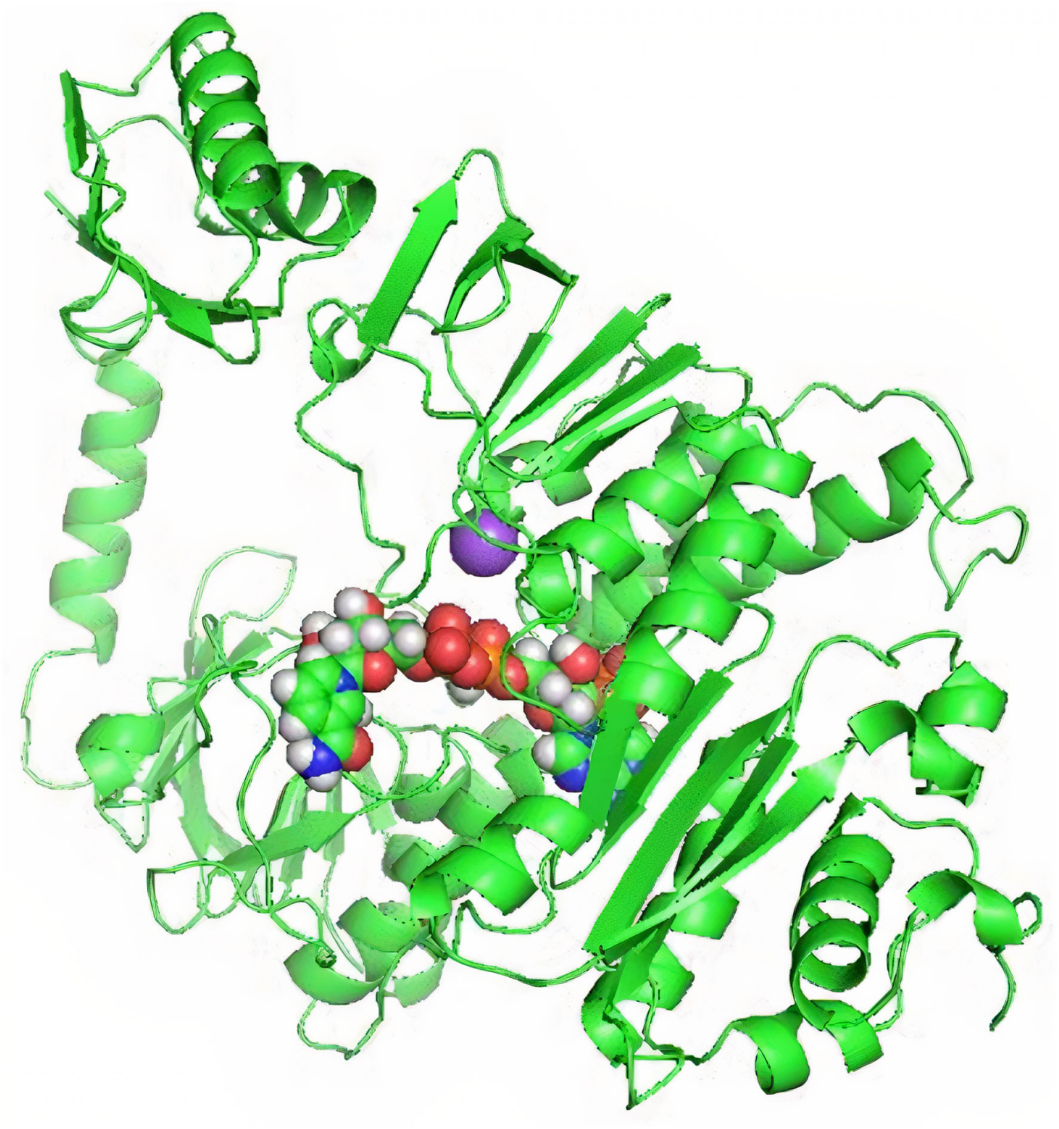
NADP^+^ docking with NADP-binding domain of MerA protein.

STRING provided the protein–protein interaction of *mer*A gene with its neighboring genes. Rhizobium bacteria were used as a model organism to study the linkages of *mer*A gene by STRING. Mercuric reductase *mer*A is the main gene responsible for degradation of toxic Hg^2+^. STRING also showed the other interacting gene like acetyltransferase component of pyruvate dehydrogenase complex (ARN20282.1), pyruvate dehydrogenase E1 (AceE), E1 component of the oxoglutarate dehydrogenase complex (SucA), glycine cleavage system protein T (GcvT), and serine hydroxymethyltransferase (GlyA). These are involved in different metabolic activities like pyruvate dehydrogenation, catabolism, transportation, and cleavage. KEGG further confirmed the role of *mer*A gene in Hd reduction. It had elaborated that *mer*A has a potential ability to oxidize metal ions by using NAD^+^ or NADP^+^ as an acceptor. Here, it also showed that Hg^2+^ also worked as a substrate for *mer*A gene activity. Figure 4 represents a 3D image of nicotinamide adenine dinucleotide phosphate (NADP^+^) domain of MerA protein docked with NADPH^+^ molecule. Interaction studies performed to check the efficiency of mercury-resistant bacteria reported that *mer*A formed complex with NADPH played a central role in Hg^2+^ vitalization. Figure 4 represents a docked complex of NADP^+^ as a substrate molecule with our MerA protein.

### Secondary Structure of MerA

Secondary structure information was investigated by computational approach. The results of PSIPRED showed different formations in MerA protein with the help of various color representations. Yellow, pink, and gray colors indicated residues forming strand, helix, and membrane interactions and transmembrane helix formations, respectively. PSIPRED also showed different polar, non-polar, and hydrophobic regions present in the protein sequence as shown in Supplementary Figure S2.

### 3D Structure of MerA Protein and Its Binding Pockets

**Figure 5:**
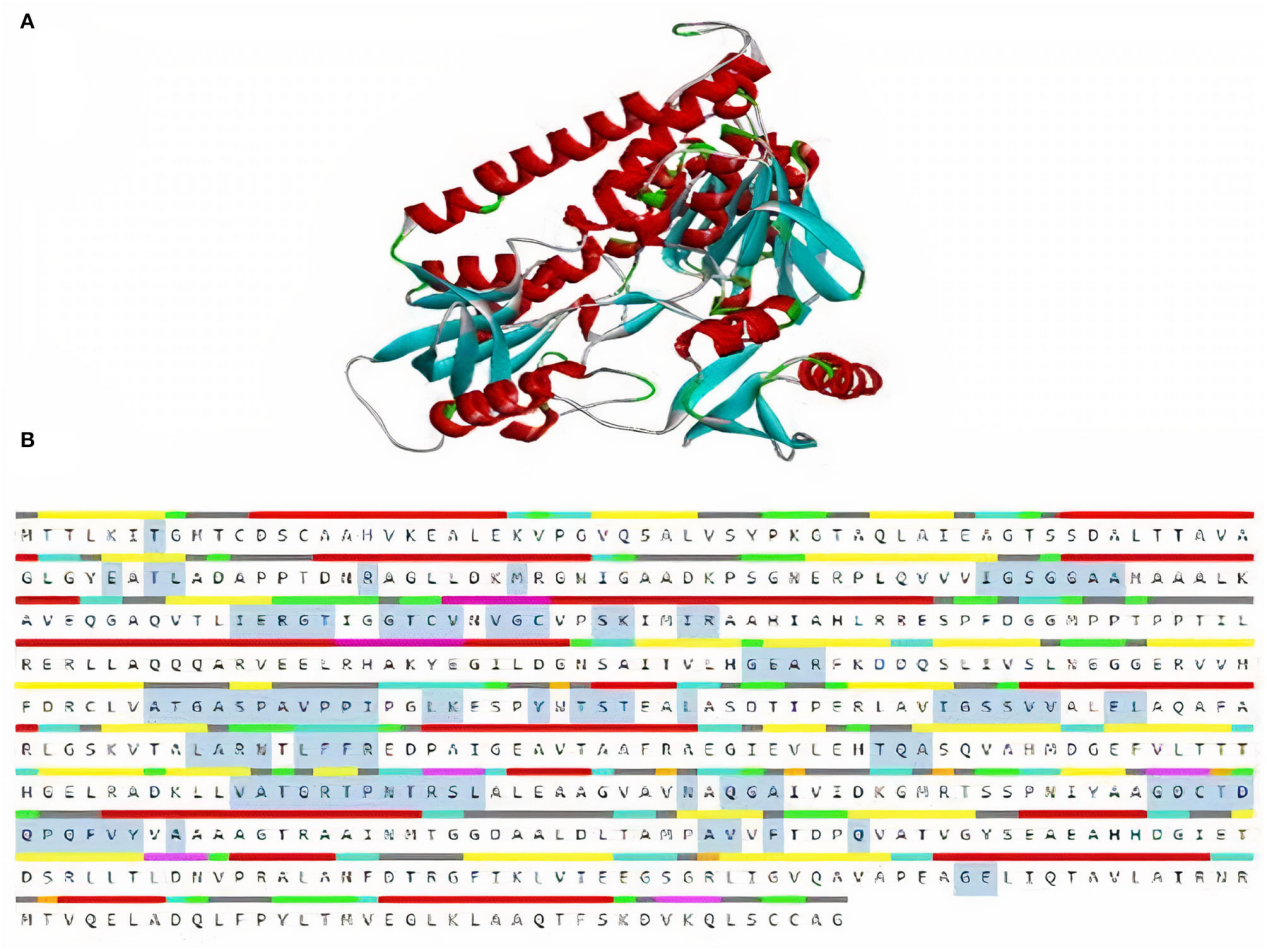
**(A)** 3D model of MerA protein. **(B)** Representation of residues in binding pockets of MerA protein.

TrRosetta was used for 3D structure prediction of MerA protein. It provided five models of protein with high confidential score (Tm: 0.822). Score higher than 0.5 T_m_ is considered to be carrying accurate predicted topology. Protein structures were predicted on the basis of sequence information and by comparing the homology of the already available structure of protein. Minimum criteria for selection of template while modeling of protein structure were as follows: confidence > 0.6, E-value <0.001, and coverage > 0.3; and values for MerA protein model templates were as follows: confidence = 100, E-value <0, and coverage > 80. Computed atlas of surface topography of proteins (CASTp) provided information regarding available binding pocket prediction of MerA protein. Highlighted sequences represented the binding pocket residues in the peptide chain as shown in Figure 5.

### Structural Validation

ERRAT verified MerA protein model and predicted the overall quality factor with value of 96.337. VERIFY 3D passed selected protein model by averaged score of 3D-1D 95.37% residues with more than 0.2. Furthermore, Ramachandran plot showed 90.7% of the residues occupied the favorable region of the graph. Remaining 9.1% residues were found in the additional favorable region and 0.2% in disallowed region of graph, and 90% residues were found in the generously allowed region of Ramachandran plot as shown in Supplementary Figure S3.

### Docking and Molecular Interactions

**Figure 6:**
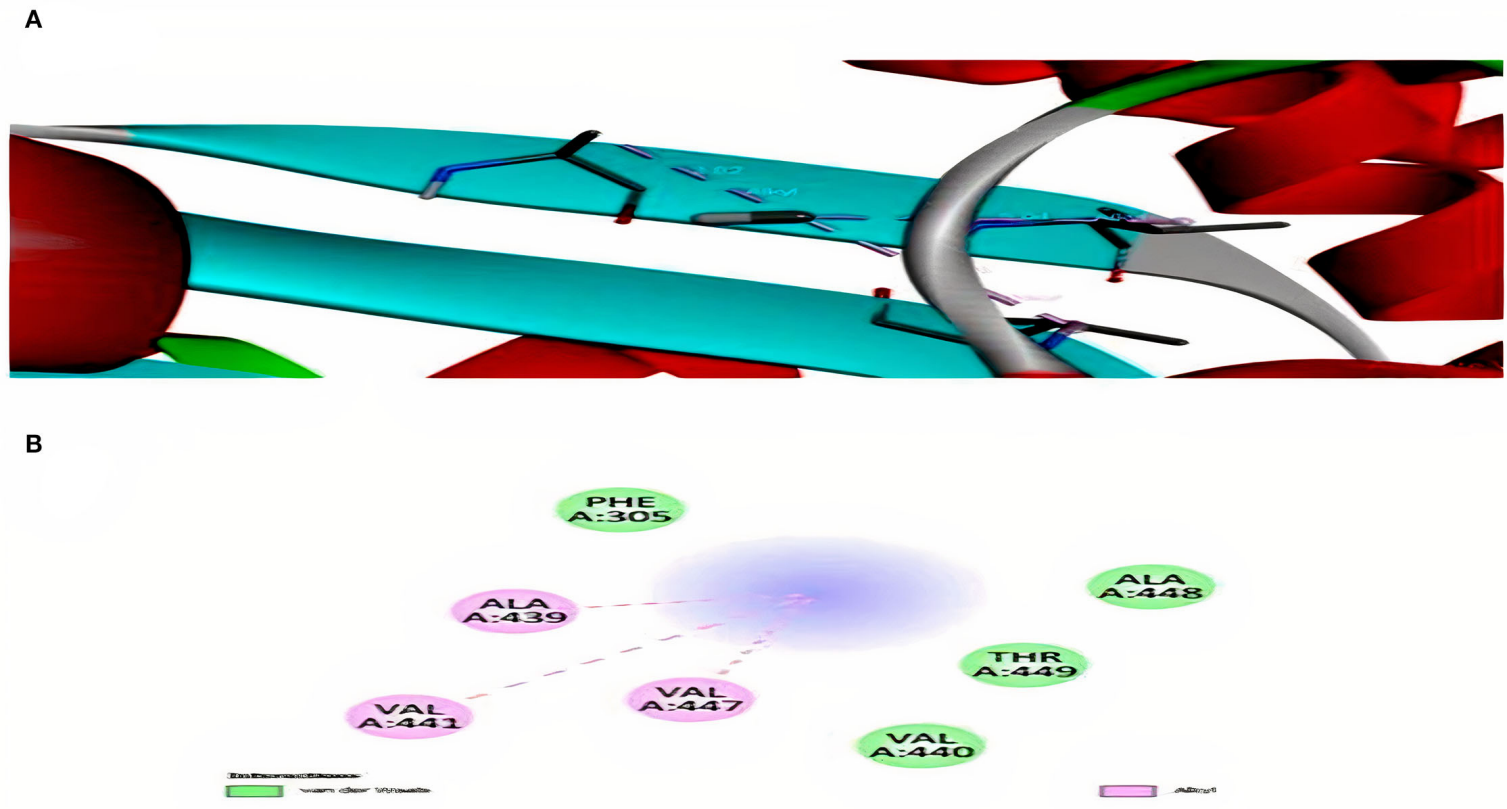
**(A)** Interaction of methyl mercury with MerA protein. **(B)** 2D representation for interaction among alanine, valine, threonine, and phenylalanine.

Interaction analysis between Hg^2+^ compounds and MerA protein explained the direct binding affinity of Hg^2+^ with MerA protein. According to the results obtained, direct affinity of Hg^2+^ toward MerA protein was made possible by the help of alkyl bonds, van der Waals forces, and hydrogen bonding. Supplementary Figure S4 and Figure 6 demonstrated the interaction of methylmercury and dimethylmercury in 3D and 2D manner. It also explained that not much strong binding had been made between the protein and Hg^2+^ directly. However, docking with complex compounds of Hg^2+^ showed a major rise in binding energy from −2.6 in methyl mercury to −7.1 in N-(Ethylmercury)-p-toluene sulfonanilide. This indicated that linkage of complex organic or inorganic group with Hg^2+^ is an essential factor in order to attain better binding energies. Moreover, structure possess by the ligand hold the potential importance whether a ligand interacts loosely or strongly with the active site (Ferreira et al., 2015). Table 3 shows the energies obtained after docking of some toxic Hg^2+^ compounds with MerA protein.

**Table 3:** Toxic compounds of mercury and their binding energies with MerA protein.

**Toxic compounds of mercury**	**Molecular formula**	**SMILES**	**Binding affinity**
Diethyl mercury	C_4_H_10_Hg	CC[Hg]CC	−3.3
Hydroxymethyl mercury	CH_3_HgOH	C[Hg^+^].[OH-]	−2.4
Methylmercury	CH_3_Hg	C[Hg]	−1.5
Mercuric chloride	HgCl_2_	Cl[Hg]Cl	−2.6
Methylmercury dicyandiamide	C_3_H_6_HgN_4_	C[Hg]N=C(N)NC#N	−4.8
Phenylmercuric acetate	C_8_H_8_HgO_2_	CC(=O)O[Hg]C1=CC=CC=C1	−6.4
Mercury selenide	HgSe	[Se]=[Hg]	−1.4
N-(Ethylmercuri)-*p*-toluene sulfonanilide	C_15_H_17_HgNO_2_S	CC[Hg]N(C_1_=CC=CC=C_1_)S(=O)(=O)C_2_=CC=C(C=C_2_)C	−7.1

## Discussion

Mercury is a naturally occurring heavy metal that poses deleterious effects on the health of living organisms (Ha et al., 2017). Mercury and its compounds are deposited in environment mainly due to the anthropogenic activities such as wastewater disposal in water bodies (Lamborg et al., 2014). All these activities have increased the concentration of mercury to an alarming extent; therefore, it is an urgent need to remove mercury and its hazardous compounds from environment by ecofriendly and cost-effective techniques (Wang et al., 2020). In recent years, the use of microorganisms gained attention to remediate mercury contaminants from environment due to its vast applications in industry (Yin et al., 2019).

Isolation of mercury-resistant bacterial species belonging to the genera of *Staphylococcus, Pseudomonas, Escherichia*, and *Bacillus* was reported in many studies (Zeyaullah et al., 2010; Keramati et al., 2011). This study was conducted to screen out mercury-resistant gram-positive bacteria *Bacillus* sp. from industrial wastewater effluents. Keeping in mind the toxic effects of Hg-pollution and the best possible options for Hg biodetoxification, Hg-resistant bacterial isolates were screened out from industrial wastewater samples and then identified by molecular characterization. Based on 16S rDNA ribotyping, selected bacterial isolates were characterized as *B. subtilis* AA-16 (OK562835), *B. cereus* AA-18 (OK562834), *Bacillus* sp. AA-20 (OK562833), *B. paramycoides* AA-30 (OK562836), and *B. thuringiensis* AA-35 (OK562837).

Detoxification of mercury by immobilized cell is a novel approach used to remediate heavy metals nowadays from the industrial wastewater (Amin and Latif, 2013). A study, conducted by Amin and Latif (2017b), reported *Candida xylopsoci* immobilized as synthetic beads in sodium alginate reduced 93% mercury while free cells remediate 75% mercury from the medium within 36 h at 30°C. In this study, *B. cereus* AA-18 showed not only high resistance to mercury but also possessed highest potential to remediate mercury from industrial wastewater. Free bacterial cells of strain AA-18 remediated 85% mercury from the medium, and its potential is enhanced to 93% when entrapped by sodium alginate. The entrapment of *B. cereus* AA-18 cells in sodium alginate enhanced the ability to remediate mercury as compared to free cells. The polymeric composition of sodium alginate entraps metal non-specifically for transporting into bacteria cells (Latif and Amin, 2017).

Immobilization poses no harmful effect on the shelf life of microorganisms instead of providing favorable environment for protecting from external harsh conditions and sustaining their genetic stability (John et al., 2011). Amin and Latif (2013) evaluated shelf life of immobilized *C. xylopsoci* with sodium alginate and showed that encapsulation has no effect on potential to reduce mercury after storage at 4°C up to 2 months. In this study, the shelf life of immobilized *B. cereus* AA-18 cells was determined at interval of four constant cycles and resulted no loss of cell viability and ability to reduce mercury was up to 90% in all cycles.

Biodetoxification of mercury is the main process by which concentration of heavy metals in wastewater is treated (Saranya et al., 2019). Perulli et al. (2021) reported the highest potential of *Pseudomonas corrugate* and *P. fluorescens* against heavy metals present in industrial wastewater. Likewise, a study conducted by Saranya et al. (2019) showed 60% of mercury removal by *B. cereus*, 95% by *B. thuringiensis*, and 68% by *B. pumilus* at 10 μg/ml concentration of HgCl_2_. In this study, a lab-scale experiment was conducted to assess the potential of *B. cereus* AA-18 cells to detoxify mercury from industrial wastewater. The percentage removal of mercury in effluent with beads was recorded 90% after 12 days. This experiment has revealed that immobilized cells never lose their potential to remediate mercury contaminant from the industrial wastewater.

In this study, the tendency of *mer*A toward Hg^2+^ biodetoxification by using the computational tools was analyzed. The significance of *mer*A gene for bacteria in order to achieve tolerance against toxic heavy metals like Hg^2+^was also investigated. It was revealed that *mer*A gene works in a network by interacting with its partner genes such as *mer*B, *mer*R, *mer*F, *mer*D, *mer*T, and *mer*P. It also interacted with some other genes that regulated different catabolic, reduction, transportation, and cleaving pathways. Among these interacting genes taking part in Hg reduction, *mer*A still holds the supreme importance. It had been seen that absence of a *mer*T or *mer*R does not necessarily affect the process of Hg^2+^ reduction to a greater extent.

According to the literature, no data provide the mechanism of Hg^2+^ reduction by Hg-resistant bacteria (Wang et al., 2020). Here, in this study, a deeper level of understanding regarding the actual interaction of Hg^2+^ and *mer*A gene was provided. It was planned to figure out that if *mer*A is responsible for reduction of Hg^2+^ stress, then it might be had strong affinity for Hg^2+^ compounds. Thus, a varying list of energy values with different compounds was obtained. This variability in energies might also be influenced by using *mer*A gene of a different bacterial strains or species. It could also study the diversity of binding affinities of *mer*A gene of different bacterial species for Hg^2+^ compounds in future.

This study included the computational analysis on taxonomic and phylogenetic distribution of *mer*A gene among bacteria species. High rate of similarity with *mer*A sequence of other bacterial species and strains of *Citrobacter* farmer, *Klebsiella michiganensis, Klebsiella pneumonia, Salmonella enteric*, and *Escherichia coli* was found. Therefore, these bacterial species will also interact in a same way with toxic Hg^2+^ compounds of environment like *mer*A gene (Dang et al., 2019). This also showed that the above bacterial species contained a same tendency to detoxify heavy metals from the environment. *Mer*A gene occupied a huge diversity of taxa which clearly indicated the importance of characterizing Hg^2+^ transformation process of much higher and diverse range of organisms (Naguib et al., 2018). The insights will definitely enhance the knowledge regarding the physiology and ecology of microbial community in modulating the form of Hg^2+^ in environment. Further exploration will lead to find out the uniqueness and potential abilities associated with the variations and evolutionary sequences of *mer*A gene.

Previous studies had not shown direct *in silico* interactions of *mer*A with toxic Hg^2+^ compounds (Asaduzzaman et al., 2019). Methylmercury is highly toxic for central and peripheral nervous system. Inhalation of Hg^2+^ vapors gives rise to neural damage, growth problems, lung impairment, microcephaly, and even death (Xia et al., 2021). This study demonstrated interactions of methylmercury with MerA protein which could become a potential source of bioremediation of methylmercury in future. Moreover, there is a need to further study to get familiar with the exact mechanism involved in bioremediation. Discovery studio tool identified the ligand bindings, active sites, and nature of bonds. The aim of visualization at molecular level was to locate accurately the position of small Hg^2+^ molecule inside MerA protein.

## Conclusion

In view of the results obtained, it is demonstrated that alginate immobilization of *Bacillus cereus* AA-18 can be a practical method for removal of mercury from industrial wastewater. Immobilized *B. cereus* AA-18 cells have exhibited an excellent potential of reusability and stability. Based on described *in vitro* and *in silico* attributes, it is concluded that *B. cereus* AA-18 is likely to be the potential candidate for biodetoxification of mercury (Hg) contaminated aquatic environment.

## Data Availability Statement

The datasets presented in this study can be found in online repositories. The names of the repository/repositories and accession number(s) can be found in the article/Supplementary Material.

## Author Contributions

AA executed the experimental work and analyzed the data. MN performed *in silico* studies of MerA protein. SR wrote the draft. AS and HS helped in executing experimental work. ZL and AB proofread the manuscript. All authors contributed to the article and approved the submitted version.

## Conflict of Interest

The authors declare that the research was conducted in the absence of any commercial or financial relationships that could be construed as a potential conflict of interest. The Reviewer MF declared a shared affiliation with the author HS at the time of the review. The Reviewer DS declared a shared affiliation with the author ZL at the time of the review.

## Publisher's Note

All claims expressed in this article are solely those of the authors and do not necessarily represent those of their affiliated organizations, or those of the publisher, the editors and the reviewers. Any product that may be evaluated in this article, or claim that may be made by its manufacturer, is not guaranteed or endorsed by the publisher.
