# Supplementary material for: In vitro and in silico Studies Reveal Bacillus cereus AA-18 as a Potential Candidate for Bioremediation of Mercury-Contaminated Wastewater
Source: Front Microbiol. 2022 Jun 6;13:847806. doi: 10.3389/fmicb.2022.847806 (PMC9207742; doi:10.3389/fmicb.2022.847806)
Supplement: Supplementary file 1 [file Data_Sheet_1.docx]

**Legends of Supplementary Figures**

**Supplementary Figure S1** Degree of conserved nature in MerA protein sequence.

**Supplementary Figure S2** Secondary structure information of MerA amino acid sequence.

**Supplementary Figure S3** Verification of protein 3D model by Ramachandran plot.

**Supplementary Figure S4** **(A)** Interactions of diethyl mercury with the MerA protein **(B)** 2D diagram showing the position and name of amino acid residues taking part in the ligand binding.

**Figure S1**


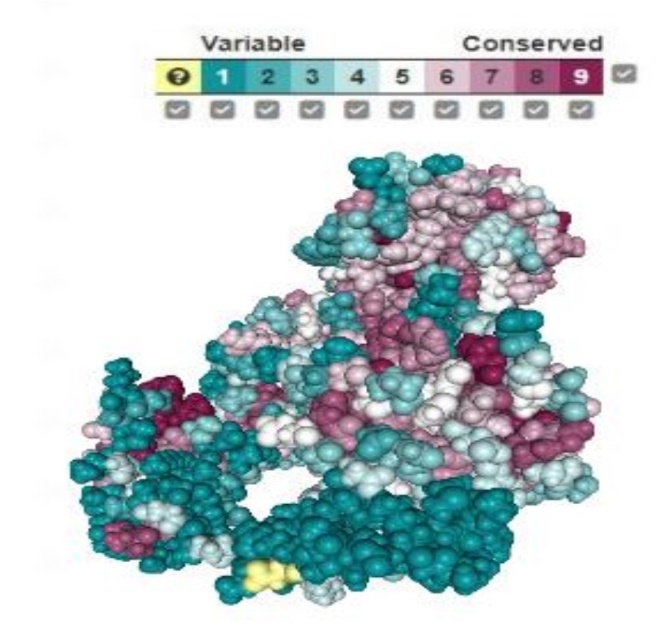


**Figure S2**


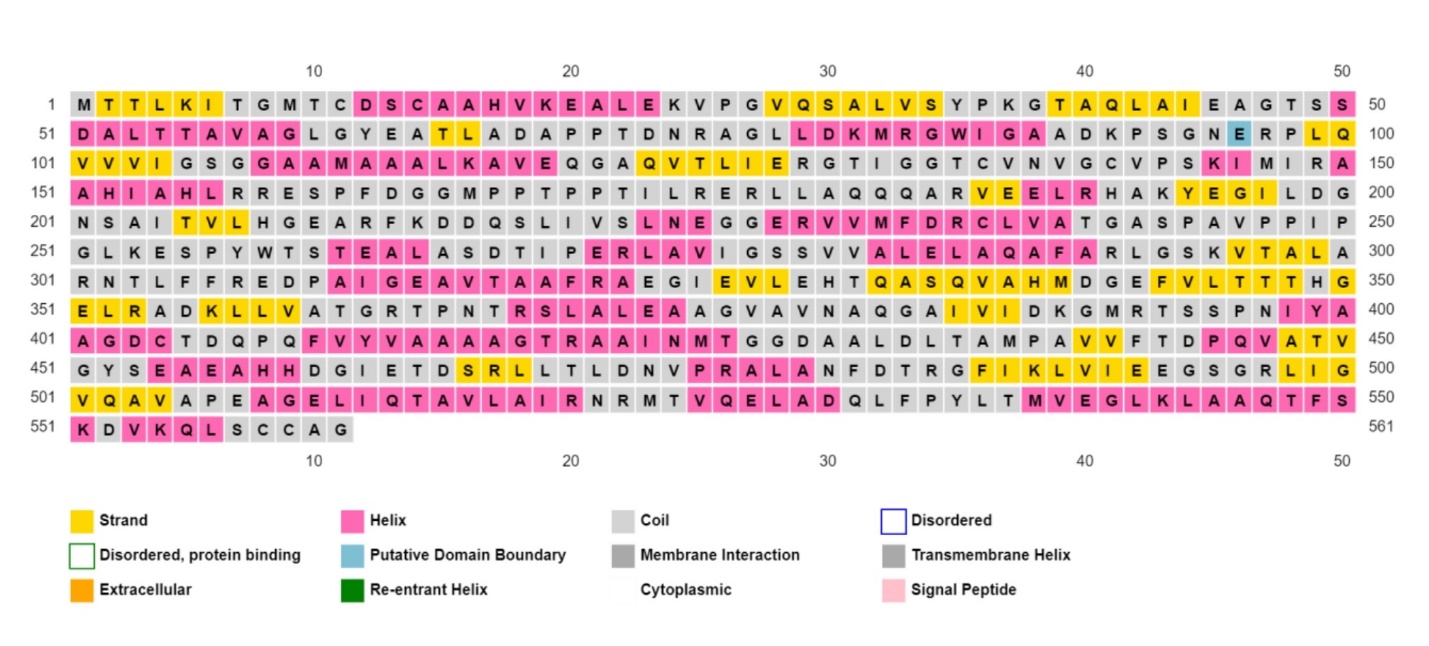


**Figure S3**

**
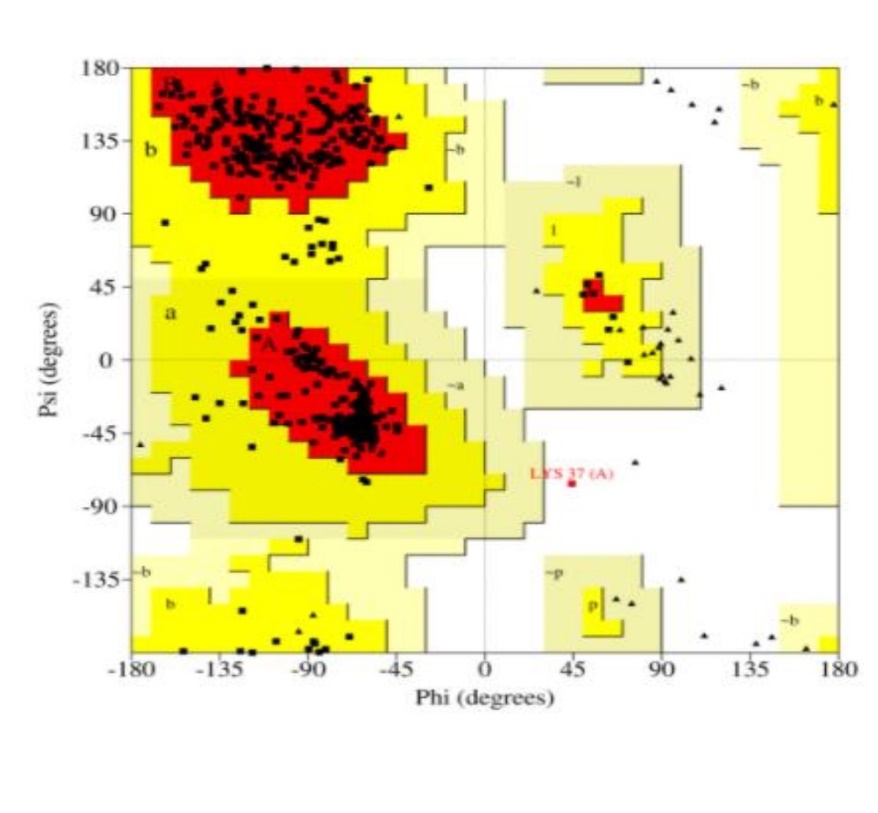
**

**Figure S4**

**
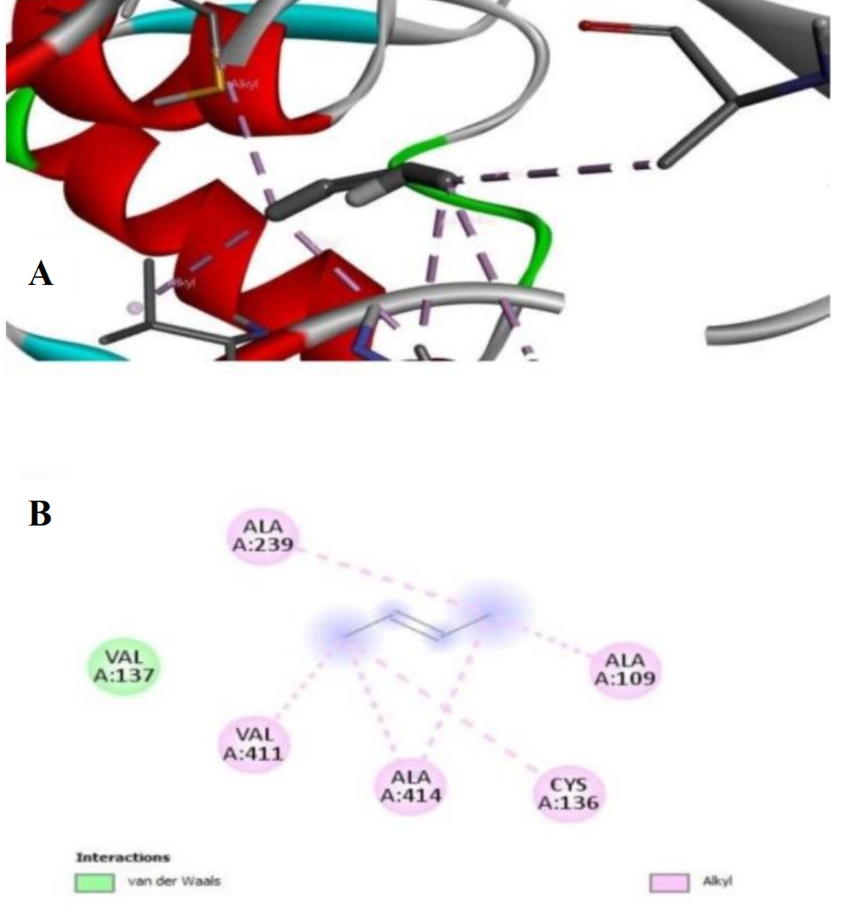
**
